# Applying Meta-Learning and Iso Principle for Development of EEG-Based Emotion Induction System

**DOI:** 10.3389/fdgth.2022.873822

**Published:** 2022-06-06

**Authors:** Kana Miyamoto, Hiroki Tanaka, Satoshi Nakamura

**Affiliations:** ^1^Division of Information Science, Nara Institute of Science and Technology, Nara, Japan; ^2^Center for Advanced Intelligence Project, RIKEN, Nara, Japan

**Keywords:** electroencephalogram (EEG), emotion induction, emotion prediction, music generation, meta-learning, iso principle

## Abstract

Music is often used for emotion induction. ince the emotions felt when listening to it vary from person to person, customized music is required. Our previous work designed a music generation system that created personalized music based on participants' emotions predicted from EEG data. Although our system effectively induced emotions, unfortunately, it suffered from two problems. The first is that a long EEG recording is required to train emotion prediction models. In this paper, we trained models with a small amount of EEG data. We proposed emotion prediction with meta-learning and compared its performance with two other training methods. The second problem is that the generated music failed to consider the participants' emotions before they listened to music. We solved this challenge by constructing a system that adapted an iso principle that gradually changed the music from close to the participants' emotions to the target emotion. Our results showed that emotion prediction with meta-learning had the lowest RMSE among three methods (*p* < 0.016). Both a music generation system based on the iso principle and our conventional music generation system more effectively induced emotion than music generation that was not based on the emotions of the participants (*p* < 0.016).

## 1. Introduction

Appropriate emotional induction is important for mental health (1–3). Many research attempts have used music to induce emotions. Even though such musical elements as rhythm and tempo induce emotions (4), not every person feels the same emotions when they listen to the same piece of music (5). In addition, the same person might experience different emotions depending on the situation. Therefore, it is challenging to induce emotions using music that takes into account the emotions of participants (6, 7). Using a subjective evaluation is a simple method for obtaining the emotions of participants. The Self-Assessment Mannequin (SAM) is often utilized for such emotional evaluation (8). However, since real-time subjective evaluations burden participants, using physiological signals has been proposed to predict emotions. Since electroencephalogram (EEG) has a high temporal resolution and are expected to be used for computer-human interaction, our work induces emotions by generating music with emotions predicted from them.

We developed a system that generates music based on participants' emotions using their EEG data (9). It consists of three elements. The first is a music generator. We treat emotions on two axes, valence and arousal, based on the circumplex model (10). The target emotion's valence and arousal to be induced are set in a range from 0 to 1 and input to a music generator, which creates music that induces an emotion similar to the inputted emotion. Note that depending on the individual differences in the feeling of an emotion and the participant's state, the input emotion and the actual emotion may not be identical. The second element is emotion prediction based on EEG. We previously showed that a convolutional neural network (CNN), which takes into account the positional relationships of EEG electrodes, effectively predicted emotions (9). The system uses a CNN to predict the participants' emotions while they listen to music in real-time. The third element is the control of a music generator. The system calculates the difference between the target emotion and the participants' emotion predicted by the EEG and adds it to the music generator's previous input. By changing the inputs of the music generator based on the participants' emotions, the system creates music that matches their characteristics. We previously verified our system that consists of these elements with six participants. We used a baseline method that generates music without considering the participants' emotions by continuously inputting the target emotion into the music generator. Our proposed method used the system to generate music from the participants' emotions in real-time. After comparing these two methods, the distance between the target emotion and the emotion that was finally induced was smaller in the proposed method, suggesting the effectiveness of the system. However, it has two problems, which we address in this paper.

The first problem is that it takes a long time to record EEG data for training the emotion prediction models because a sufficient amount of EEG data is required to train them. In our experiment, the EEG data were recorded for only 30 min, considering the burden placed on the participants. Because of this time factor, we trained an emotion prediction model using EEG data for 30 min and used the system on a different day. Even though the EEG recording time must be shortened to improve the system's usability, a lack of EEG data negatively impacts model training (11). Previous studies solved this problem by proposing transfer learning (9, 12), which adapts a pre-trained model from one domain to another (13). With a small amount of EEG data to retrain a pre-trained model which was trained on a large amount of EEG data, more accurate predictions can be made than with just a small amount of EEG data. However, in previous studies, the pre-training model mixed the EEG data of multiple people and treated them as one big amount of data (9, 12). Perhaps individual EEG characteristics cannot be taken into account because no individuals are recognized. Meta-learning, which is an effective solution to this problem, has been used for few-shot learning, fast many-shot optimization, robustness to domain-shift, and so on (14). It helps models acquire experience through multiple tasks with which to improve future learning performance. There are gradient-descent, reinforcement learning, and evolutionary search as its optimizer. Some previous studies on EEG predictions trained models with one person's EEG data as a single task and demonstrated the effectiveness of meta-learning. Model-agnostic meta-learning (MAML) (15) is one popular type of meta-learning with gradient-descent. MAML trains an initial model that easily adapts to any task from multiple tasks. Therefore, the initial model can adapt to new tasks with a small amount of data. Previous studies predicted sleep levels (16) and motor imagery (17) using EEG with MAML. MAML was also used for emotion prediction using EEG, and its effectiveness was investigated using the DEAP dataset with music video stimuli and the SEED dataset with movie stimuli (18). However, we use only music, which is an auditory stimulus. Since the type of stimulus influences emotion elicitation (19), we believe that the effectiveness of applying MAML must be tested for emotion prediction while listening to music. We have two baseline methods: one trains a model using multiple participants' EEG data without MAML, and the other trains a model with a small amount of a single participant's EEG data. We compared the emotion prediction performance of the proposed method with two baseline methods and constructed an emotion induction system using a model trained with MAML.

The second problem is that the music generator creates music without identifying the participants' emotions before they listen to it. We showed that the inputs to the music generator and the emotions felt by the participants are similar. Therefore, we tried to increase/decrease the music generator's inputs based on the participants' emotions using empirically-determined formulas. Here is an example of a case where we want to induce a high valence. First, a high valence, which is the target emotion, is input into the music generator. The participants listen to the generated music. If they experience a low valence, the next valence input will be higher, and the music generator will try to induce a higher valence. As shown above, we proposed a method that makes music that rapidly moves a participants' emotion toward the target emotion, starting from the beginning of listening to a piece of music, and adjusts the music generator's inputs based on their states. The proposed method more effectively induced emotions than the baseline method in which the target emotion is continuously input to the music generator. In music therapy, the iso principle, which is used in emotion induction (20, 21), plays music that is close to the participant's emotion and gradually leads them to the target emotion. In a previous study (22), participants with sadness listened to two pieces of music: sad music or happy music. The results showed that listening to happy music after sad music induced more positive emotions. Our previously proposed method rapidly induced emotions to target emotions without considering the emotions of the participants before they listened to music. In this paper, we develop a system based on this iso principle and investigate whether music generation with it and our conventional music generation effectively induce emotions.

Our paper provides the following two contributions:

1. It verifies EEG-based emotion prediction using meta-learning.
2. It evaluates an emotion induction system using meta-learning and the iso principle.

This paper is an extension of our previously proposed emotion induction system (9). To improve it, we utilized emotion prediction with the meta-learning of our previous work (23) and newly investigated the relationship between the amount of training data and the performance of models trained by meta-learning. We also adopted the iso principle as a new music generation method and evaluated a new emotion induction system that applied meta-learning and the iso principle.

## 2. EEG Emotion Prediction With Meta-Learning

In Section 2, we train a highly accurate emotion prediction model with a small amount of a participant's EEG data while listening to music. First, we describe the EEG dataset and the features and the model structure for training models. We then introduce our proposed method using meta-learning and two baseline methods and emphasize its effectiveness by comparing the performances of the three methods.

### 2.1. Dataset

In our previous work, we created a dataset containing EEG data and subjective evaluations of emotions felt by participants while they listened to music (9). The experiment was approved by the Ethics Committee of the Nara Institute of Science and Technology. Its participants were 10 males and 10 females. The music was created using a music generator designed based on a previous study (6) that made music that induced emotions similar to its valence and arousal inputs. We created 41 pieces of music by inputting 41 various emotions into the music generator. The detailed input values are described in our previous work (9). The sample music can be heard here: https://sites.google.com/view/music-generator. We used a 3-s EEG before listening to the music for a baseline correction based on a previous study (24). The participants silently gazed at a cross mark in the center of the monitor for 5 s for the baseline correction because the initial silent state may contain body movement noise. They then listened to a 20 s piece of music while continuing to gaze at the cross mark. In studies using music to induce emotion, using 30–60 s of music is appropriate (25). However, we tried to record EEG using a variety of music. To incorporate the burden on the participants who wore the electroencephalograph, the music was set to 20 s, referring to previous studies (6, 26). After listening to the music, they evaluated the valence they felt using SAM on a 9-point scale between 0 and 1 and then evaluated their arousal in the same manner. This procedure was repeated for all 41 pieces of music. The EEG data were recorded using a Quick-30 headset manufactured by CGX.

### 2.2. Features and Model Structure

**Figure 1:**
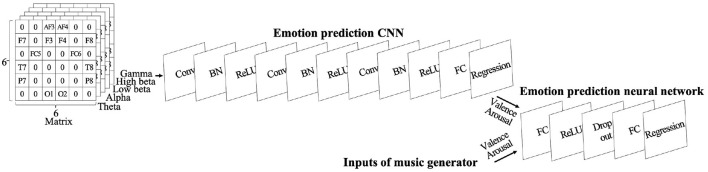
Model structure: Upper part is a CNN that predicts emotion from EEG data. Right part is a neural network that uses predicted emotions from CNN and music generator's inputs for emotion prediction.

For each piece of music, we recorded 5 s of EEG data before they listened to the music and 20 s while they listened. We used the last 3 s before listening for a baseline correction and divided these 23 s of EEG data into 1 s pieces without overlap. Then we used band-pass filters and divided them into five frequency bands: theta (4–7 Hz), alpha (8–13 Hz), low beta (14–21 Hz), high beta (22–29 Hz), and gamma (30–45 Hz). We calculated the logarithms of the variances of the EEG waveforms as features and subtracted the average feature values of the three samples before listening to the music from each feature of the 20 samples for baseline correction. Although Quick-30 provides 29 EEG channels, emotion prediction using a selection of 14 EEG channels provided higher performance in our previous work (9). We also used 14 EEG channels in this paper and calculated 20 samples of features with a total of 70 dimensions per piece of music. The features calculated as described above were mapped to matrices, shown in the upper left corner of Figure 1. The matrices took into account the position of the EEG channels and the characteristics of the frequency bands. The grid is 6 × 6 × five matrices. The areas without electrodes are embedded with zeros.

We used a CNN for the emotion prediction using EEG. The structure is shown in Table 1 and at the top of Figure 1. We used an SGD optimizer.

**Table 1:** Structures of CNN and neural network: Conv is convolutional layer, BN is batch normalization layer, FC is fully connected layer, and Drop is drop-out layer.

**Model**	**Layer**	**Kernel**	**Channels**	**Stride**	**Drop-out rate**
CNN	Conv+BN+ReLU	2×2	8	1	–
	Conv+BN+ReLU	2×2	8	1	–
	Conv+BN+ReLU	2×2	8	1	–
	FC	–	2	–	–
Neural network	FC+ReLU+Drop	–	8	–	0.2
	FC	–	2	–	–

### 2.3. Emotion Prediction Methods

We compared the following three methods for predicting emotions using a small amount of EEG data while listening to music:

- Method A: multiple participants' EEG with MAML;
- Method B: multiple participants' EEG without MAML;
- Method C: a single participant's EEG.

We set one participant as a target. Methods A and B were trained by the pre-training models with/without MAML using the EEG data of multiple participants. The pre-trained models were trained without the target participant's EEG data. Then the pre-training models were fine-tuned by the target participant's EEG data. Method C was trained with just the target participant's EEG data.

#### 2.3.1. Method A: Multiple Participants' EEG With MAML

**Figure 2:**
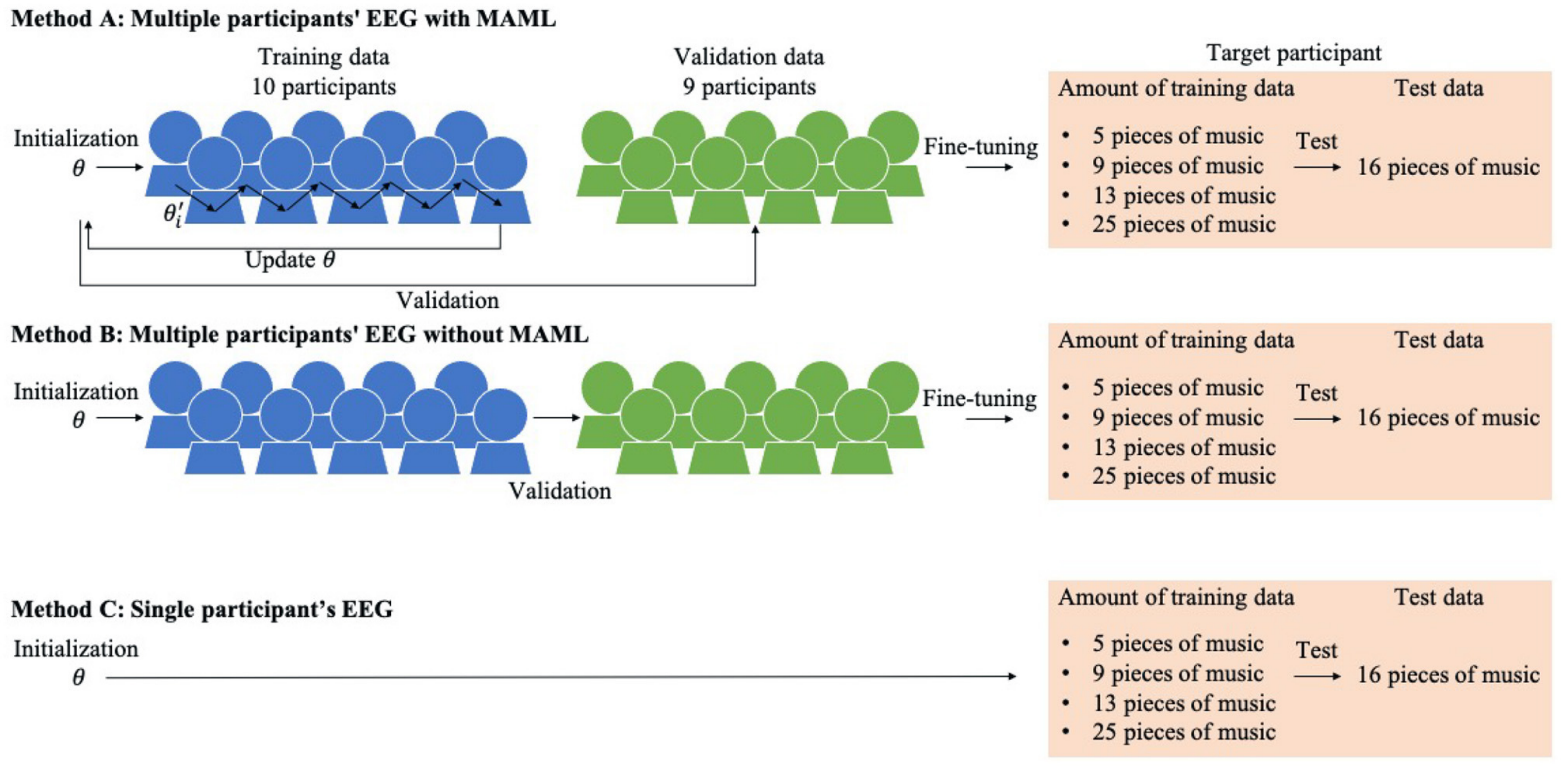
Three emotion prediction methods using a single target participant's small amount of EEG data.

We first describe method A, which is our proposed scheme. We used Algorithm 1, and the training procedure is shown at the top of Figure 2.

**Algorithm 1:** MAML for emotion prediction using EEG data.

**Require:** p(T): distribution over tasks
**Require:** α, β: learning rate
1: Randomly initialize θ
2: Sample training tasks $T_{i}\sim p(T)$
3: **for** each *iteration* **do**
4: **for** each $T_{i}$ **do**
5: Select data of 20 pieces of music $D_{i}=\left\{x,y\right\}$ from $T_{i}$
6: Evaluate $\nabla_{\theta }{ℒ}_{T}{}_{i}(f_{\theta })$ using $D_{i}$ and ${ℒ}_{T}{}_{i}$
7: Update parameters: $\theta_{i}^{\prime }=\theta -\alpha \nabla_{\theta }{ℒ}_{T}{}_{i}(f_{\theta })$
8: Select data of about 21 pieces of music $D_{i}^{{}^{\prime }}=\left\{x^{{}^{\prime }},y^{{}^{\prime }}\right\}$ from $T_{i}$
9: **end for**
10: Update $\theta \leftarrow \theta -\beta \nabla_{\theta }\sum_{T_{i}\sim p(T)}L_{Ti}(f_{\theta_{i}^{\prime }})$ using each $D_{i}^{\prime }$ and $L_{Ti}$
11: **end for**

For pre-training, we randomly extracted 10 participants' data from our dataset. We considered one participant's data as one task and selected the data of 20 pieces of music from each task *i*. We set EEG data *x* and labels *y* of the emotions felt by the participant as support set $D_{i}=\left\{x,y\right\}$ and EEG data *x*′ and labels *y*′ of the remaining 21 pieces of music as query set $D_{i}^{{}^{\prime }}=\left\{x^{{}^{\prime }},y^{{}^{\prime }}\right\}$. We first used initialized model parameters θ and updated them using the support set in each task. These updated parameters were evaluated with the query set, and the loss was calculated in each task. After all the tasks were computed, model parameters θ were updated to minimize the loss for all of them. This process was repeated. The hyperparameter sets were α ∈ {10^−1^, 10^−2^}, and β = 10^−1^. The number of hyperparameters was small because we needed to train 20 pre-trained models for each target participant to reduce the computation time. We used all of the data from the remaining nine participants in the dataset and set them as the validation data. The hyperparameters were determined using the validation data. The model was trained until the validation loss did not decrease for five consecutive iterations.

We fine-tuned our pre-trained model using the target participant's data. To reduce the preparation time of using the emotion induction system, a small amount of data must be collected from the participants before the emotion induction. Therefore, we examined how much to reduce the amount of data for fine-tuning. We prepared four different kinds of training data to investigate the relationship between the amount of training data and the model performance. We created the music used for the training data by inputting the following emotions into the music generator: five pieces of music ({val,aro}={0,0}; {0,1}; {0.5,0.5}; {1,0}; {1,1}), nine pieces of music (five pieces of music + {val,aro}={0,0.5}; {0.5,0}; {0.5,1}; {1,0.5}), 13 pieces of music (nine pieces of music + {val,aro}={0.25,0.25}; {0.25,0.75}; {0.75,0.25}; {0.75,0.75}), and 25 pieces of music (13 pieces of music + {val,aro}={0,0.25}; {0,0.75}; {0.25,0}; {0.25,0.5};{0.25,1};{0.5,0.25};{0.5,0.75};{0.75,0};{0.75,0.5};{0.75,1};{1,0.25};{1,0.75}). We selected these music generator inputs to be taken uniformly on the valence and arousal coordinates. The learning rate was set to 0.1 and the iterations to 13. These parameters were determined based on our previous work (23). The fine-tuned model was evaluated using 16 pieces of music, which were not included in the training data.

#### 2.3.2. Method B: Multiple Participants' EEG Without MAML

Next we describe method B, which is a baseline method whose training procedure is shown in the middle of Figure 2.

For pre-training, we randomly extracted the data of 10 participants from our dataset. Multiple participants were regarded as one large amount of data. Initial parameters θ were trained with a batch size of 1,024 using the data. The hyperparameter sets were *learning rate* ∈ {10^−1^, 10^−2^}. We used all of the data from the remaining nine participants in the dataset and set them as the validation data from which the hyperparameters were determined. The model was trained until the validation loss did not decrease for five consecutive iterations. The pre-trained model was fine-tuned using the target participant's data. We prepared four different kinds of training data to investigate the relationship between the number of training data and the performance as well as the proposed method. The learning rate was set to 0.1 and the iterations to 10. We evaluated the fine-tuned model using the test data as well as the proposed method.

#### 2.3.3. Method C: Single Participant's EEG

Next we describe method C, which is a baseline method whose training procedure is shown at the bottom of Figure 2. It has no pre-training. The model was trained from initial parameters θ by the same procedure as in the fine-tuning of the proposed method and the other baseline method. We prepared four different kinds of training data to investigate the relationship between the amount of training data and the performance as well as the other methods. The learning rate was set to 0.1 and the iterations to 25. The fine-tuned model was evaluated using the test data like the other methods.

### 2.4. Comparison of Three Methods for Predicting Emotions Using EEG

**Figure 3:**
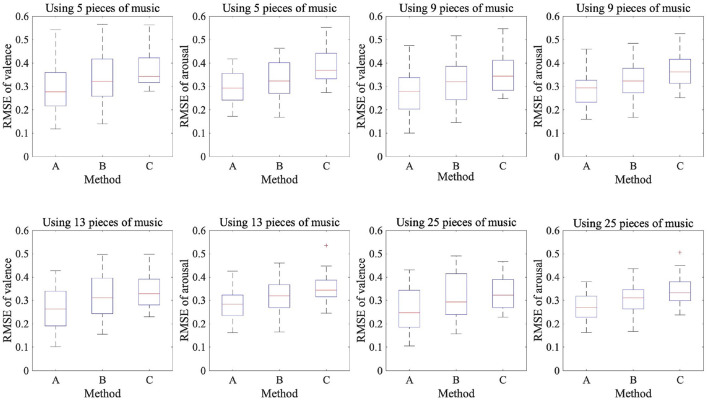
Box plots of 20 participants' RMSEs of felt and predicted emotions using EEG data.

We trained 20 models with different target participants in each method. The emotion prediction results are shown in Table 2 and Figure 3, which show the RMSE between the label values of the dataset and the values predicted by the CNN. We found a significant difference among the three methods using the same amount of data in both valence and arousal in the Friedman test (*p* < 0.05). We used Wilcoxon signed-rank tests with Bonferroni correction to compare the three methods. In the valence results, there was a significant difference between methods A and B and methods A and C with any amount of data (*p* < 0.016). However, for methods B and C, there was a significant difference when nine pieces of music were used (*p* < 0.016). In the arousal results, there was a significant difference between methods A and B, between methods A and C, and between methods B and C with any amount of data (*p* < 0.016). Proposed method A had a significantly lower RMSE than the two baseline methods for both valence and arousal. We also found a significant difference in the RMSE trained by four different training data amounts of proposed method A of both valence and arousal in the Friedman test (*p* < 0.05). The results indicated that the performance of the emotion prediction depended on the amount of training data.

**Table 2:** Participants' mean and standard deviation of RMSEs of felt and predicted emotions using EEG data: Bold indicates RMSE of proposed method with a significant difference from baseline methods.

**Method**	**5 pieces**		**9 pieces**		**13 pieces**		**25 pieces**	
	Val	Aro	Val	Aro	Val	Aro	Val	Aro
A	0.298	0.298	0.275	0.290	0.262	0.285	0.256	0.274
	(0.121)	(0.071)	(0.101)	(0.071)	(0.096)	(0.066)	(0.098)	(0.058)
B	0.347	0.328	0.325	0.323	0.318	0.320	0.312	0.308
	(0.122)	(0.082)	(0.103)	(0.080)	(0.099)	(0.077)	(0.101)	(0.067)
C	0.378	0.391	0.355	0.366	0.338	0.354	0.331	0.344
	(0.080)	(0.079)	(0.084)	(0.068)	(0.071)	(0.070)	(0.070)	(0.069)

These results showed that in the case of arousal prediction with a small amount of EEG data while listening to music, methods A and B had lower RMSE than method C. Moreover, method A had a lower RMSE than method C for the prediction valence. In the case of using multiple participants' EEG data, method A had a lower RMSE than method B in the predictions of both the valence and arousal. Furthermore, the RMSE was lower when the amount of training data was larger in proposed method A, indicating that the amount of training data is important for highly accurate emotion prediction.

### 2.5. Predicting Emotions Using EEG and Music Generator Inputs

Our previous work argued that a neural network using emotions predicted from EEG and a music generator's inputs can predict participants' emotions with high performance (9). Since the music generator makes music to induce emotions that resemble its inputs, we considered its inputs the predicted emotions felt by the participants when they listened to music. We also used an emotion prediction neural network in this paper to stabilize the predictions by using two types of information as its inputs: the emotion predicted by the CNN with MAML using EEG and the music generator's inputs. We compared the prediction performance of the following two models:

- Model A: CNN
- Model B: CNN + neural network.

The neural network's structure is shown in Table 1 and in the right part of Figure 1. We used an SGD optimizer, fine-tuned the CNN pre-trained by MAML, and trained the neural network using the target participant's data. CNN's fine-tuning method was identical as in Section 2.3.1. The learning rate was set to 0.1 and the iterations to 100 for training the neural network.

**Figure 4:**
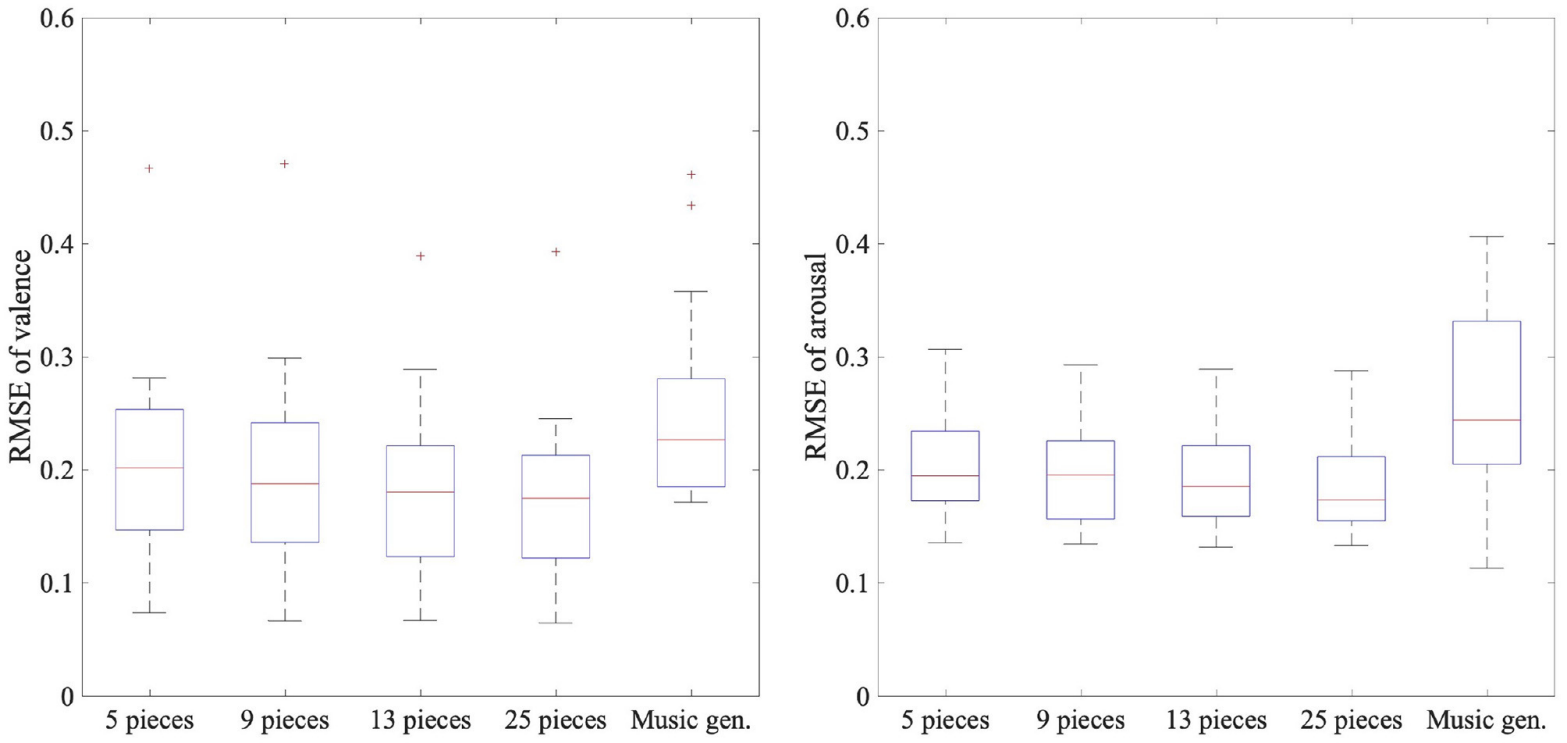
Box plots of 20 participants' RMSEs of felt and predicted emotions using EEG data and music generator's inputs: Music gen. indicates emotion prediction using music generator's inputs.

The emotion prediction results are described in Table 3 and Figure 4, which show the RMSE between the label values of the dataset and the predicted values using model B or the music generator's inputs of each target participant. Compared with Table 2, we found a significant difference among the following three predictions using the same amount of data in both the valence and the arousal in the Friedman test (*p* < 0.05): using model A, model B, and the music generator's inputs. We used Wilcoxon signed-rank tests with Bonferroni correction to compare the three predictions. In the valence results, there was a significant difference between models A and B when any amount of data was used (*p* < 0.016). For model B and using the music generator's inputs, there was a significant difference when 13 or more pieces of music were used (*p* < 0.016). In the arousal results, there was a significant difference between models A and B when any amount of data was used (*p* < 0.016). For model B and using the music generator's inputs, there was a significant difference when nine or more pieces of music were used (*p* < 0.016). We also found a significant difference in the RMSE trained by four different amounts of training data of model B in both the valence and the arousal in the Friedman test (*p* < 0.05).

**Table 3:** Participants' mean and standard deviation of RMSEs of felt and predicted emotions using EEG data and music generator's inputs: Music gen. indicates emotion prediction using music generator's inputs.

**Model B: CNN + neural network**								**Music gen**.	
**5 pieces**		**9 pieces**		**13 pieces**		**25 pieces**	
**Val**	**Aro**	**Val**	**Aro**	**Val**	**Aro**	**Val**	**Aro**	**Val**	**Aro**
0.202	0.204	0.194	**0.196**	**0.181**	**0.192**	**0.171**	**0.184**	0.251	0.258
(0.088)	(0.044)	(0.093)	(0.043)	(0.078)	(0.041)	(0.075)	(0.039)	(0.084)	(0.086)

*Bold indicates RMSE with a significant difference from both predictions using model A and music generator's inputs*.

These results showed that for emotion prediction with a small amount of EEG while listening to music, model B had lower RMSE than model A. Furthermore, model B had lower RMSE than using the music generator's inputs in the predictions of both the valence and arousal when 13 or more pieces of music were used. The RMSE values were lower when the amount of training data was larger in model B. This result indicates that the amount of training data is important for highly accurate emotion prediction, as in the results of Table 2.

In this section, we experimentally used a small amount of EEG data while our participants listened to music to train the emotion prediction models. The results showed that MAML was effective for emotion prediction. We also developed a neural network using the emotions predicted by a CNN trained by MAML and the music generator's inputs. A neural network using both the EEG data and the music generator's inputs improved the performance of the emotion prediction. In the next section, we construct and validate an emotion induction system using the CNN trained by MAML and a neural network as an emotion prediction model.

## 3. Emotion Induction Using Models Trained by Meta-Learning

**Figure 5:**
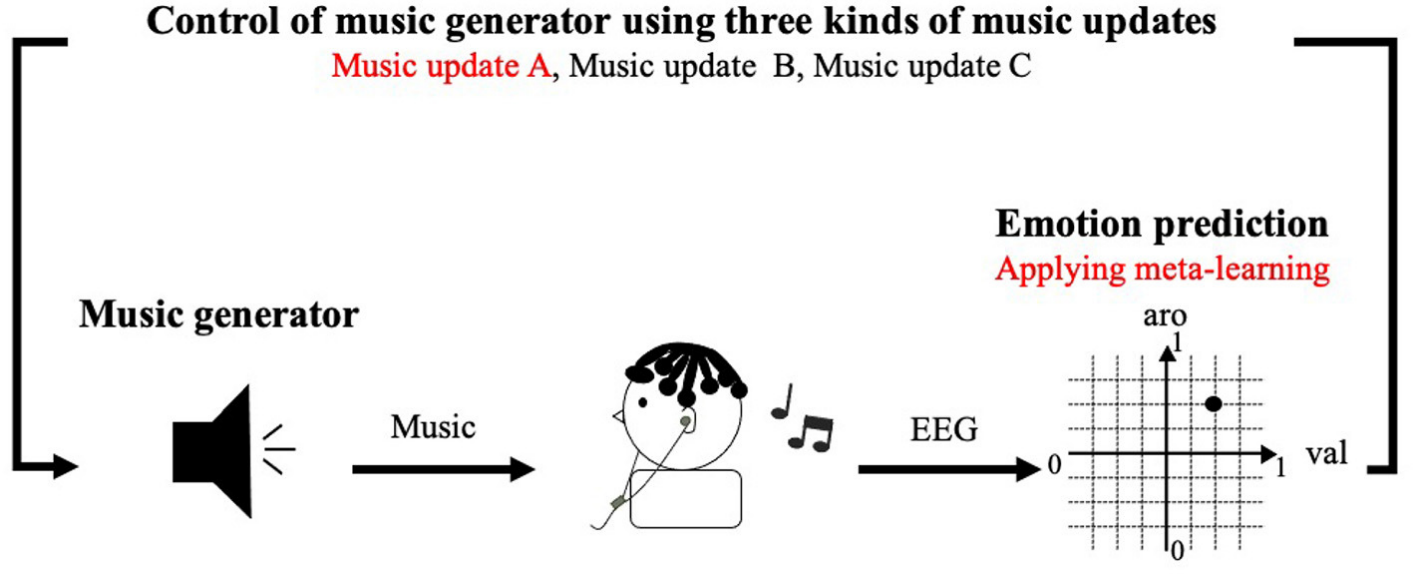
Emotion induction system using meta-learning: Red text is newly implemented methods in this paper.

In Section 3, we construct an emotion induction system using a CNN trained by MAML and a neural network for emotion prediction (Figure 5). Since the music generation method used in the conventional system ignored the emotions of the participants before they listened to music, we developed a music generation method based on the iso principle. Our system generates music that resembles a participant's emotion before he listened to music and gradually generated music that was close to the target emotion. We investigated whether a system using meta-learning and the iso principle effectively induced emotion.

### 3.1. Utilization of Emotion Induction System

We used our system into which we embedded an EEG-based emotion prediction model trained by MAML to generate music in real-time. We next present information on the data collection during emotion induction.

#### 3.1.1. Participants

Ten healthy people (age: 26.6 years; eight males, two females) participated in this experiment, which was approved by the ETHICS Committee of the Nara Institute of Science and Technology. They did not participate in the previous experiments described in Section 2. We used Section 2's dataset to train a pre-training model for the emotion prediction. If the same participant's data were used for pre-training and fine-tuning, we believe that the emotion prediction performance might be distorted by using the same participant's data for pre-training and fine-tuning. For this reason, we carefully recruited these participants.

#### 3.1.2. Target Emotions

We set the following five types of target emotions to be induced in the participants: {val,aro} = {0.125,0.125}; {0.125,0.875}; {0.5,0.5}; {0.875,0.125}; {0.875,0.875}. Although in our previous work we set nine target emotions (9), here we reduced them to five due to experimental time limitations. These five emotions were taken from the nine target emotions of our previous work.

#### 3.1.3. Pre-training Model

In Section 2, we showed that training a CNN with MAML predicted emotions best when using a small amount of EEG data. Therefore, we used MAML to train the pre-training CNN with EEG data. We used our dataset with 10 participants for the training data and 10 for the validation data and tuned the learning rate and the iterations. We fine-tuned the pre-training model with the data of a target participant who joined the experiment in Section 3. The neural network's effectiveness is also shown in Section 2 using the emotions predicted by the CNN and the music generator's inputs. We only trained the neural network with the target participant's data.

#### 3.1.4. Experimental Protocol

At the experiment's beginning, the participants wore earphones at a desk with a monitor and listened to five 15 s samples with the following input values to the music generator: {val,aro}={0,0};{0,1};{0.5,0.5};{1,0};{1,1}.

**Figure 6:**
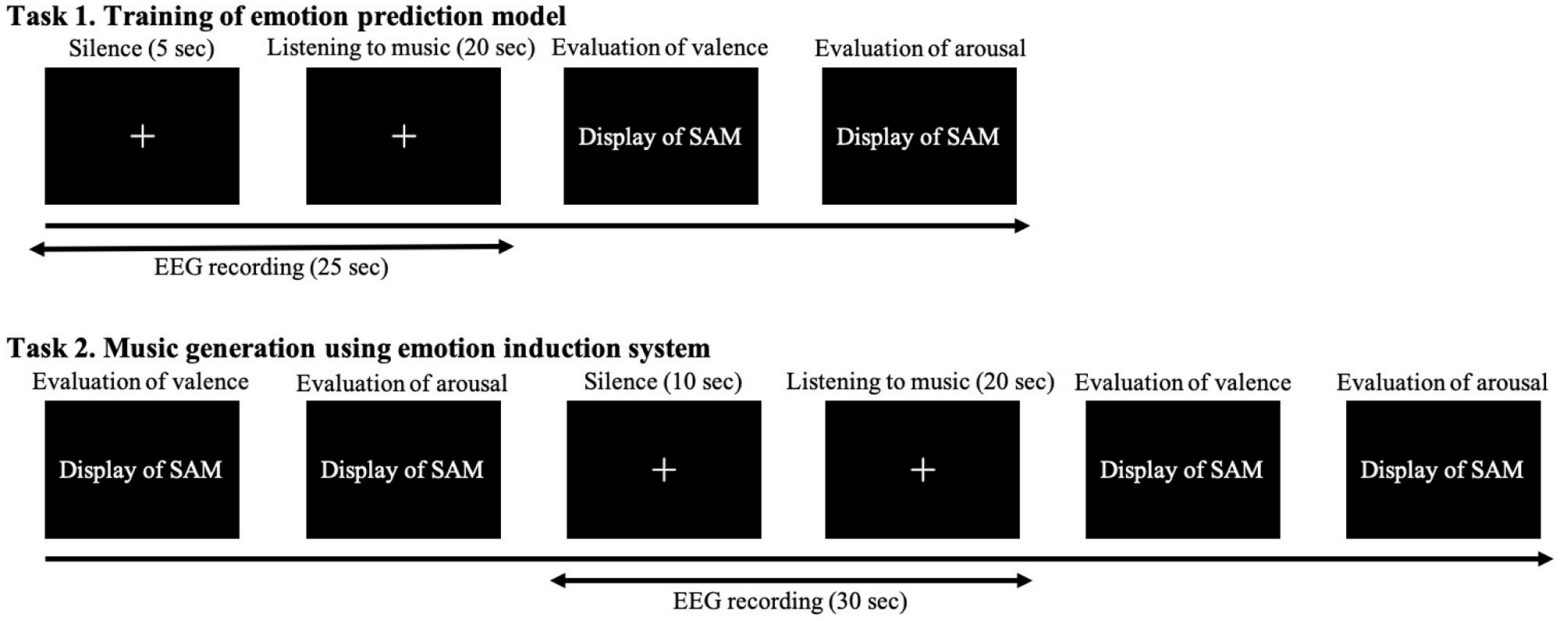
Experimental protocol.

Then we conducted a practice session. In this experiment, we trained the models for predicting the participants' emotions using a pre-training model in task 1 and induced emotions by generating music in a system embedded with the models in task 2. The details of each experiment are shown in Figure 6. Our participants practiced each task once to understand how to perform both tasks. First, we introduce task 1, which trained the models for predicting the participants' emotions. They silently gazed at a cross mark in the center of the monitor for 5 s and then listened to each 20 s music sample while continuing to gaze at the cross mark. After listening to the music, they separately evaluated their emotions using SAM on a 9-point scale from 0 to 1 for valence and arousal. They practiced the experiment with one of two pieces of music: {val,aro}={0.125,0.25} or {0.875,0.75}. Next we introduce task 2, which is the emotion induction procedure of music generation in the system. Before listening to the music, our participants separately evaluated their emotions using SAM on a continuous value from 0 to 1 for valence and arousal. They again silently gazed at the cross mark for 10 s and listened to each 20 s music sample that has 20 measures while continuing to gaze at the cross mark. After listening, they evaluated their emotions using SAM; then they took a 10 s break. They practiced the experiment with one of two pieces of music: {val,aro}= {0.875,0.75} or {0.125,0.25}.

After the practice, the participants put on a Quick-30 headset manufactured by CGX. We repeated the same procedures from the practice session to record the EEG data and the subjective evaluations of their experienced emotions while they listened. Section 2 showed how the emotion prediction's performance improved with more training data. We used the 13 pieces of music to record the EEG data and the subjective evaluations so that the participants continued to wear the electroencephalograph for <30 min. Next we fine-tuned the pre-training model using the recorded data. The preprocessing method is the same as described in Section 2. The learning rate was set to 0.1 and the iterations to 13 for fine-tuning model A's CNN. The learning rate was set to 0.1 and the iterations to 100 for training model B's neural network. Then we conducted emotion induction by music generation for the system embedded with the model in task 2. The participants listened to 15 pieces of music using three different music generation methods. Each method generated music that was intended to invoke five target emotions. EEG data from 2 to 5 s after the onset of silence were used as a baseline correction. The emotion before listening to music was predicted using the EEG data from 5 to 6 s after the onset of silence just using model A. Emotions while listening to music were predicted once every four measures using model B. For this prediction, we used a 1 s EEG after the beginning of the first measure in four measures. The EEG's sampling frequency in the whole experiment was 100 Hz, and the tools used in the experiment included MATLAB (2021b), Lab Streaming Layer, Psychtoolbox (8, 27, 28), Cakewalk, and LoopBe1.

### 3.2. Music Update Methods

We applied the following three methods for the music updates using Algorithm 2:

- Music update A: music updates with the iso principle;
- Music update B: music updates without the iso principle;
- Music update C: fixed music without participants' emotions.

**Algorithm 2:** Update music generator's inputs.

1: Record 1 s EEG during the silent state
2: Predict emotion before listening to music using EEG
3: **if** Music update A **then**
4: Set a music generator's inputs as a participant's emotion before listening to music
5: **else if** Music update B or C **then**
6: Set a music generator's inputs as a target emotion
7: **end if**
8: **for** each *update* **do**
9: Start generating music using the music generator's inputs
10: Record a 1 s EEG
11: Predict the current emotion using EEG
12: **if** Music update A **then**
13: Update the music generator's inputs using formulas (2) and (4)
14: **else if** Music update B **then**
15: Update the music generator's inputs using formulas (5) and (6)
16: **else if** Music update C **then**
17: Update the music generator's inputs using formulas (7) and (8)
18: **end if**
19: **end for**

#### 3.2.1. Music Update A: Music Update With Iso Principle

Neither method from our previous work took into account the participants' emotions before they listened to music (9). However, the iso principle showed that using music that is close to the participant's emotion at the beginning and gradually changing it to induce the target emotion effectively induces emotion. In this method, the music generator's inputs were changed based on the iso principle once every four measures using participant's emotion and determined by the following formulas:


(1) $$mid\_target_{\text{val}}(s+1)=\{\begin{array}{ll} pred_{\text{val}}(s) & if\;s=0,\\ pred_{\text{val}}(0)+s*(target_{\text{val}}-pred_{\text{val}}(0))/(s_{max}-1) & if\;0<s<s_{max}. \end{array}$$

(2) $$input_{\text{val}}(s+1)=\{\begin{array}{ll} mid\_target_{\text{val}}(s+1) & if\;s=0,\\ mid\_target_{\text{val}}(s+1)+0.5*(mid\_target_{\text{val}}(s)-pred_{\text{val}}(s)) & if\;0<s<s_{max}. \end{array}$$

(3) $$mid\_target_{\text{aro}}(s+1)=\{\begin{array}{ll} pred_{\text{aro}}(s) & if\;s=0,\\ pred_{\text{aro}}(0)+s*(target_{\text{aro}}-pred_{\text{aro}}(0))/(s_{max}-1) & if\;0<s<s_{max}. \end{array}$$

(4) $$input_{\text{aro}}(s+1)=\{\begin{array}{ll} mid\_target_{\text{aro}}(s+1) & if\;s=0,\\ mid\_target_{\text{aro}}(s+1)+0.5*(mid\_target_{\text{aro}}(s)-pred_{\text{aro}}(s)) & if\;0<s<s_{max}. \end{array}$$

**Figure 7:**
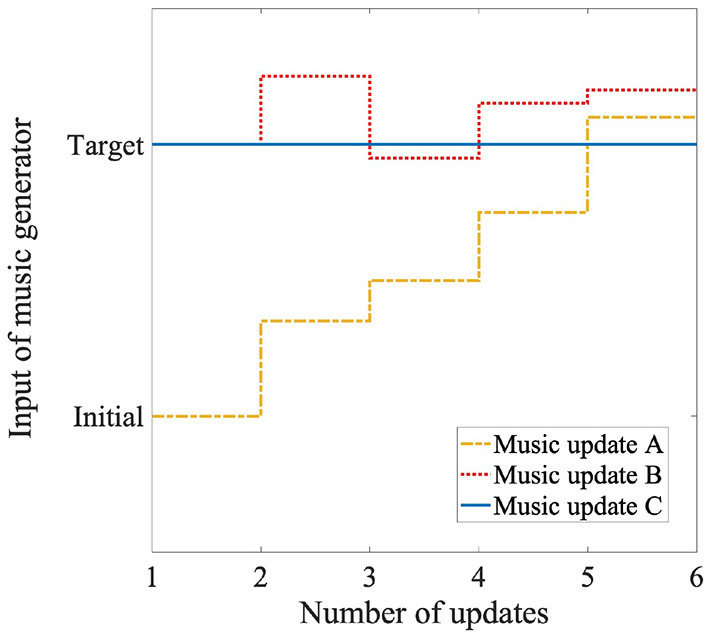
Conceptual scheme of control of music generator using three methods: Target denotes target emotion for emotion induction. Initial denotes participant's emotion before listening to music.

In the formulas, *s* represents the number of times the inputs are updated, *s* = 0 denotes the period before the music generator starts making music, and *s* = 1 denotes when the music generator starts making music. Updates were made up to *s* = 5. *s*_*max*_ represents the number of times the music was updated, and *s*_*max*_ = 5. *input* represents the input emotion to the music generator, *target* represents the target emotion in the induction, *mid*_*target* represents the intermediate target emotion determined by the number of times the music was updated, and *pred* represents the emotion predicted from the EEG while listening to music. First, the system predicts the participant's emotion before listening to the music using only model A. The difference between the target and predicted emotions was divided by four, which is the maximum number of times the inputs to the music generator were updated using the participant's emotion; the intermediate target emotion was set for each update. In the first loop, the participant's emotion before listening was input directly to the music generator. In the next loop, the system added half of the difference between the intermediate target emotion and the participant's emotion predicted by model B to the next intermediate target emotion. We used a half value because the music generator's inputs were between 0 and 1 for both the valence and arousal. Inputs outside the range were set to 0 or 1. If the difference value is large, the music generator will continue to receive a constant input, such as 0 or 1, and the music will not change. For these reasons, half of this difference was added. In this way, the system generated music that gradually induced emotions while taking into account how the participants were feeling. We show a conceptual scheme of the music generator's control in the yellow dotted line (Figure 7).

#### 3.2.2. Music Update B: Music Update Without Iso Principle

In this method, the system first created music by inputting the target emotion into the music generator and adjusting the inputs once every four measures using the participant's emotion. The inputs were determined by the following formulas:


(5) $$input_{\text{val}}(s+1)=\{\begin{array}{ll} target_{\text{val}} & if\;s=0,\\ input_{\text{val}}(s)+0.5*(target_{\text{val}}-pred_{\text{val}}(s)) & if\;0<s<s_{max}. \end{array}$$

(6) $$input_{\text{aro}}(s+1)=\{\begin{array}{ll} target_{\text{aro}} & if\;s=0,\\ input_{\text{aro}}(s)+0.5*(target_{\text{aro}}-pred_{\text{aro}}(s)) & if\;0<s<s_{max}. \end{array}$$

First, the system predicted the emotion of the participants before they listened, although the predicted emotion was not used for the music generation. In the first loop, the target emotion was input directly to the music generator. In the next loop, the system added half of the difference between the target and the predicted emotions of the participant to the previous inputs of the music generator. In this way, the system generated music that rapidly induced emotions while taking into account how the participants felt. We show a conceptual scheme of the music generator's control in the red dotted line in Figure 7.

#### 3.2.3. Music Update C: Fixed Music Without Participants' Emotions

In this method, the system kept inputting the target emotion to the music generator. The inputs were determined by the following formulas:


(7) $$\begin{array}{l} input_{\text{val}}\left(s\right)=target_{\text{val}}if0<s<s_{max}. \end{array}$$

(8) $$\begin{array}{l} input_{\text{aro}}\left(s\right)=target_{\text{aro}}if0<s<s_{max}. \end{array}$$

The system predicts the emotions of the participants before they listened to the music and while they listened to it, although the predicted emotions were not used for music generation. We show a conceptual scheme of the control of the music generator in the blue line in Figure 7.

### 3.3. Evaluation of Emotion Induction System

We fine-tuned the emotion prediction model of the emotion induction system for each participant in this experiment described in Section 3. The model's performance is important because two music generation methods used the emotions predicted by it. We first confirmed the performance of the trained emotion prediction models of the 10 participants. The emotion prediction results are shown in Table 4, which shows the RMSE between the label values evaluated by the participants of their emotions and the predicted values by models A or B before/after listening to all the music. As a reference of the conventional system, the following are the means of the RMSE of the emotion predictions after listening to music with model B for all the participants: valence: 0.201 and arousal: 0.180. The conditions of the conventional system and the current system are different: the number of participants and target emotions, the structure of the emotion prediction model, the emotion evaluation method, and the length of silence before listening to the music. Therefore, comparing the conventional and current systems is impossible. However, from the conventional system's results as a reference, no large difference seems to exist in the RMSE of emotion prediction.

**Table 4:** RMSE of felt and predicted emotions before or after listening to music in current system: Bold indicates performance of CNN and neural network used by system to generate music.

	**Before listening to music**		**After listening to music**			
**Par**.	**Model A**		**Model A**		**Model B**	
	**Val**	**Aro**	**Val**	**Aro**	**Val**	**Aro**
1	0.183	0.117	0.159	0.162	0.144	0.193
2	0.170	0.317	0.259	0.198	0.126	0.155
3	0.117	0.203	0.297	0.329	0.265	0.311
4	0.163	0.196	0.221	0.239	0.168	0.164
5	0.301	0.199	0.458	0.379	0.321	0.191
6	0.200	0.183	0.233	0.163	0.132	0.163
7	0.251	0.264	0.490	0.358	0.200	0.225
8	0.317	0.167	0.318	0.322	0.253	0.326
9	0.190	0.248	0.192	0.210	0.152	0.155
10	0.242	0.203	0.332	0.356	0.196	0.200
Mean	0.213	0.210	0.296	0.272	**0.196**	**0.208**
SD	0.063	0.055	0.109	0.086	0.065	0.062

We also investigated the effect of emotion induction by the system. We evaluated the emotion induction performance by calculating the distance between the target emotion and the final predicted emotion by model B using following the formula:


(9) $$\begin{array}{l} distance=\sqrt{{\left(target_{\text{val}}-pred_{\text{val}}\left(s_{max}\right)\right)}^{2}+{\left(target_{\text{aro}}-pred_{\text{aro}}\left(s_{max}\right)\right)}^{2}}. \end{array}$$

The calculated means of the distances of the five types of emotional induction are shown in Table 5. In the conventional system of our previous work, the following are the means of the distances for all the participants: music update B: 0.248 and music update C: 0.296. The results showed that both the current and conventional systems effectively induced emotions by taking into account the participants' emotions.

**Table 5:** Distance between target and induced emotions: Bold indicates distance with a significant difference from baseline method.

**Par**.	**Music update A**	**Music update B**	**Music update C**

1	0.483	0.496	0.450
2	0.399	0.371	0.401
3	0.387	0.445	0.348
4	0.318	0.345	0.418
5	0.385	0.353	0.378
6	0.299	0.296	0.393
7	0.234	0.190	0.362
8	0.267	0.284	0.318
9	0.309	0.287	0.340
10	0.224	0.303	0.338
Mean	**0.331**	**0.337**	0.375
SD	0.082	0.087	0.041

**Figure 8:**
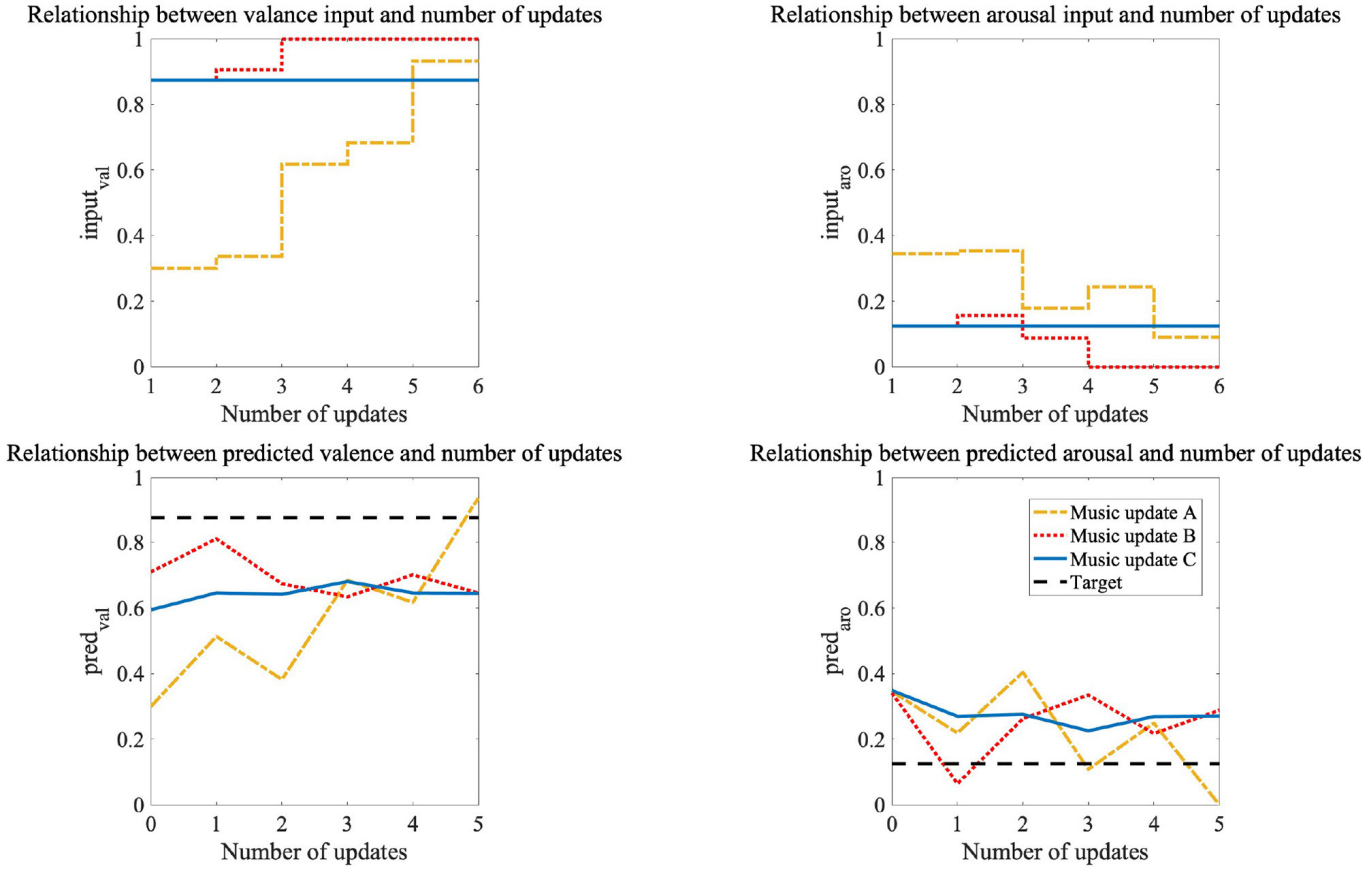
Plots of inputs of music generator and emotions predicted from CNN and neural network in participant eight: Target emotion is {val,aro} = {0.875,0.125}.

We not only compared the current system with the conventional one but also the performances of the three methods in the current system. We found a significant difference among them in the Friedman test using the distances calculated for all the pieces of music for all the participants (*p* < 0.05). We used Wilcoxon signed-rank tests with Bonferroni correction for comparisons of the three methods. There was a significant difference between music updates A and C and between music updates B and C (*p* < 0.016). From the above results, we conclude that music updates A and B, which generated music according to the participants' emotions, more effectively induced emotions than music update C that didn't generate music according to their emotions. However, we found no significant difference between music updates A and B. We show plots of the music generator's inputs and the emotions predicted from model B in Figure 8. This is the result for participant eight; the target emotion is {val,aro} = {0.875,0.125}, and music update A provided more effective emotion induction than the other two methods. The number of updates is zero before listening to music, and the music generator created music from five updates. Music updates B and C suddenly generated music that induced the target emotion, and music update A generated music that gradually induced the target emotion, starting from music close to the participant's emotion before listening to the music. Music update A led to an emotion closer to the target than the other two methods (Figure 8).

## 4. Conclusion and Future Works

Our conventional emotion induction system using music and EEG suffered from two problems. It took a long time to record EEG to train the emotion prediction model, which is a required step for constructing our system. The second problem was that the music generator's control method created music without the participants' emotions before they listened to music. We solved these problems by developing a new system that uses meta-learning and the iso principle. To solve the first problem, we proposed a meta-learning method using a small amount of EEG data while listening to music. The proposed method predicted emotions with higher performance than the baseline methods without meta-learning. In addition, the system into which the trained model with meta-learning was embedded effectively induced emotions. Therefore, we conclude that meta-learning reduced the EEG recording time and increased the usability of our emotion induction system.

To solve the second problem, our system induced emotions through music generation using the iso principle. The methods with/without it, which took the participants' emotions into account, more effectively induced emotions than the methods that did not consider them. We found no significant difference between the methods with/without the iso principle. In previous studies on it, emotions opposite to the target emotion were induced in the participants beforehand, and then the participants were led to the target emotion (22). In our experiment, we did not induce emotions opposite to the target emotion before our participants listened to music. We believe that music generation with the iso principle may be more effective than the other two music generation methods when the participants are induced to the target emotion from an opposite emotion. We set the length of the music sample to 20 s. The results are limited in terms of the music duration. We need to consider how many seconds of music to use for more effective emotion induction in the future.

Our future works will investigate two problems. The first is to improve meta-learning for more efficient emotion prediction. Meta-learning has been actively studied in recent years, and improvements are being developed (29, 30). Improvements in meta-learning that address the EEG characteristics will raise the accuracy of emotion prediction. The second problem is the investigation of more diverse music generation methods. We used predefined formulas to control the music generator. In the future, we will develop a method using deep learning to control it based on the participants' characteristics.

## Data Availability Statement

The datasets presented in this article are not readily available because the datasets contain some private information. Requests to access the datasets should be directed to KM, miyamoto.kana.mk4@is.naist.jp.

## Ethics Statement

The studies involving human participants were reviewed and approved by the Ethics Committee of the Nara Institute of Science and Technology (reference number: 2019-I-23). The patients/participants provided their written informed consent to participate in this study.

## Author Contributions

KM, HT, and SN designed and conducted the experiment. KM wrote the manuscript. SN is the project's principal investigator and directs all the research. All authors contributed to the article and approved the submitted version.

## Funding

This work was supported by JST CREST Grant Number JPMJCR19A5, Japan and the RIKEN Junior Research Associate Program.

## Conflict of Interest

The authors declare that the research was conducted in the absence of any commercial or financial relationships that could be construed as a potential conflict of interest.

## Publisher's Note

All claims expressed in this article are solely those of the authors and do not necessarily represent those of their affiliated organizations, or those of the publisher, the editors and the reviewers. Any product that may be evaluated in this article, or claim that may be made by its manufacturer, is not guaranteed or endorsed by the publisher.
